# Interdisciplinary Successful Revascularization of Traumatic Occlusion of the Right Common Carotid Artery

**DOI:** 10.7759/cureus.55395

**Published:** 2024-03-02

**Authors:** Boris Ilchev, Vasil Chervenkov, Nikolay Valchev, Vladimir Nakov, Tsvetan Minchev, Georgi Vassilev, Tsvetomir Tsvetanov, Lili Laleva, Milko Milev, Toma Spiriev

**Affiliations:** 1 Department of Vascular Surgery, Acibadem City Clinic University Hospital Tokuda, Sofia, BGR; 2 Department of Neurosurgery, Acibadem City Clinic University Hospital Tokuda, Sofia, BGR; 3 Department of Thoracic Surgery, Acibadem City Clinic University Hospital Tokuda, Sofia, BGR; 4 Department of Cardiac Surgery, Acibadem City Clinic University Hospital Tokuda, Sofia, BGR; 5 Department of Angiology, Acibadem City Clinic University Hospital Tokuda, Sofia, BGR

**Keywords:** traumatic vessel occlusion, blunt carotid artery injury, 3d visualization, photogrammetry scanning, vascular surgery

## Abstract

Blunt carotid artery injury (BCI) poses a rare yet severe threat following vascular trauma, often leading to significant morbidity and mortality. We present a case of a 33-year-old male who suffered complete thrombotic occlusion of the right common carotid artery (CCA) following a workplace accident. Clinical evaluation revealed profound neurological deficits, prompting multidisciplinary surgical intervention guided by the Denver criteria (Grade I - disruption inside the vessel that results in a narrowing of the lumen by less than 25%; Grade II - dissection or intramural hematoma causing greater than 25% stenosis; Grade III - comprises pseudoaneurysm formation; Grade IV - causes total vessel occlusion; Grade V - describes vessel transection with extravasation). Surgical exploration unveiled extensive arterial damage, necessitating thrombectomy, primary repair, and double-layered patch angioplasty using an autologous saphenous vein. Postoperative recovery was uneventful, with the restoration of pulsatile blood flow confirmed by Doppler ultrasound. Three-month follow-up demonstrated patent arterial reconstruction and improved cerebral perfusion, despite the persistent neurological deficits. Our case underscores the challenges in diagnosing and managing BCI, advocating for a tailored approach based on injury severity and neurological status. While conservative management remains standard, surgical intervention offers a viable option in select cases, particularly those with complete vessel occlusion and neurological compromise. Long-term surveillance is imperative to assess the durability of arterial reconstruction and monitor for recurrent thromboembolic events. Further research is warranted to refine management algorithms and elucidate optimal treatment strategies in this rare but critical vascular pathology.

## Introduction

Blunt carotid artery injury (BCI) is an uncommon yet severe issue in vascular trauma. Despite its rarity, BCI is linked with mortality rates ranging from 20% to 40% [1]. Additionally, it leads to substantial severe neurological complications in as many as half of the individuals who survive the initial trauma [2]. Injuries to the carotid artery may lead to dissection, formation of an intramural thrombus, or pseudoaneurysm, resulting in reduced blood flow to the brain and increased risk of thromboembolic events [3]. However, common carotid artery (CCA) lesions after blunt injuries to the neck are extremely rare [4-6]. These carotid injuries can lead to serious consequences with elevated rates of both mortality and morbidity, often exhibiting delayed manifestations [1,7-9]. The spectrum of treatment options spans from solely using antithrombotic therapy to surgical intervention [1]. The latter is considered in cases of pseudoaneurysm, unsuccessful or contraindicated medical treatment, and worsening of neurological symptoms [1]. However, the best strategy, encompassing the treatment method and timing, remains a subject of ongoing discussion [9-12]. We hereby present a case of successful multidisciplinary surgical treatment after a complete thrombosis of the right common carotid artery caused by blunt compressive damage to the artery.

## Case presentation

We report a case of a 33-year-old male who was admitted to our institution a month after experiencing a workplace accident. The patient was compressed against a wall by a truck, resulting in loss of consciousness and partial memory loss.

Upon admission, the patient presented with symptoms of vision loss in the right eye and decreased vision in the left eye. He also exhibited coordination deficits, necessitating the use of a cane for mobility, and reported weakness and paresthesia in the fingers of his left hand. The patient had sustained a contusion of the chest and abdomen, followed by a pneumothorax, for which he underwent multiple surgical interventions in another medical facility, which was the main reason for his late presentation in our hospital. At the time of arrival, he was on a single antithrombotic agent-clopidogrel. No other comorbidities were present at this point.

Clinical findings

A neurological examination revealed loss of vision in the right eye, without perception of light and pupillary reflex. The left eye showed a mild decrease in vision. The patient exhibited decreased muscle power in the distal musculature of the left hand and leg (3/5 Manual Muscle Testing (MMT)), as well as coordination deficits in the lower legs.

Diagnostic assessment

**Figure 1:**
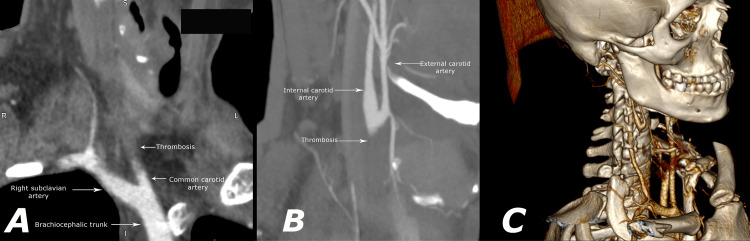
А and B) Preoperative CT angiogram (CTA) showing complete occlusion of the right common carotid artery with decreased collateral filling of the right internal carotid and middle cerebral artery; C) 3D reconstruction based on the CTA providing volumetric representation of the data

A CT angiography revealed a complete traumatic occlusion of the common carotid artery starting near its origin, a collateral decreased filling of the right internal carotid artery (ICA), and the right middle cerebral artery, both of which had a significantly decreased diameter compared to their contralateral arteries (Figure 1). The CT scan also described ischemic changes in the right brain hemisphere. A Doppler sonography, which showed a decreased volume flow in the right middle cerebral artery and no Doppler signal in the right internal carotid, was performed.

Therapeutic intervention

Taking into consideration the decreased volume flow and a grade IV Denver scale blunt carotid injury, а multidisciplinary surgical team was assembled consisting of a vascular surgeon, neurosurgeon, and thoracic surgeon, and a decision was made to perform a surgical intervention of the right common carotid artery.

**Figure 2:**
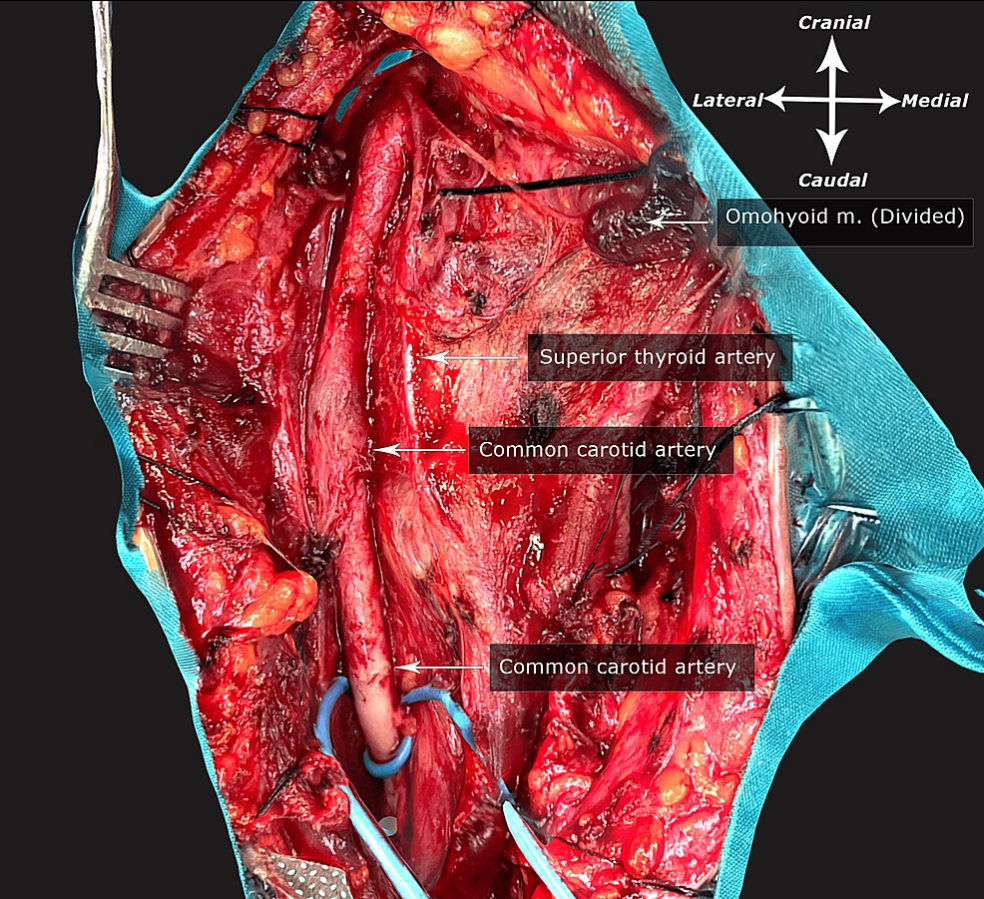
Initial surgical neck dissection presenting the common carotid artery and the occlusion site 3D model of the initial surgical access created by the authors available at: https://sketchfab.com/3d-models/carotid-dissection-78f3ad82e781432baf33adce6abbba65

The patient was positioned in the supine posture with extension and leftward rotation of the head. Initially, a traditional surgical approach was employed to access the carotid bifurcation, involving a 10-12 cm incision along the medial surface of the sternocleidomastoid muscle. Sequentially traversing layers of adipose tissue and the platysma muscle, a meticulous dissection was performed, reaching the carotid sheath. After the careful incision of the sheath, the carotid bifurcation was meticulously exposed (Figure 2).

**Figure 3:**
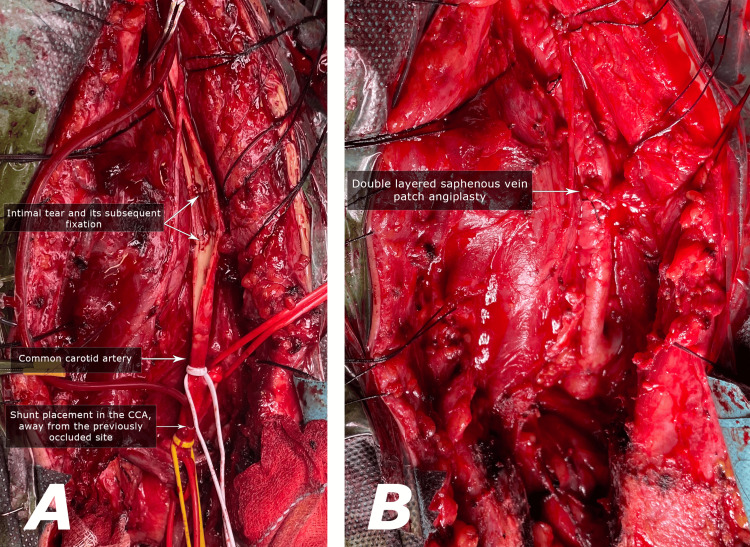
A) A thoracic surgeon executed a midline thoracic incision and an upper ministernotomy, revealing the brachiocephalic trunk, subclavian artery, and the remainder of the common carotid artery. A longitudinal arteriotomy was performed alongside the occluded region, followed by the introduction of a three-lumen Pruitt-Inahara shunt. Thrombosis within the common carotid artery, accompanied by an underlying intimal flap is presented. B) A direct thrombectomy was executed, and the intimal flap was fixated with 6-0 nylon sutures. The arteriotomy was subsequently closed using a double-layered autologous saphenous vein patch.

Throughout the dissection within the common carotid region, post-traumatic tissue changes were encountered, necessitating an extension of the surgical access. To address this, a thoracic surgeon executed a midline thoracic incision and an upper ministernotomy, revealing the brachiocephalic trunk, the subclavian artery, and the remainder of the common carotid artery (Figure 3A).

Intraoperative Doppler ultrasonography was employed to confirm the occluded segment, commencing approximately 1.5 cm beyond the common carotid origin and extending proximally to the carotid bifurcation. Utilizing vascular loops and under the protection of 5000 IU of heparin, the common, internal, and external carotid arteries were individually clamped in a standard fashion.

A three-lumen Pruitt-Inahara shunt was introduced utilizing the Seldinger technique for the distal CCA, and a small surgical incision was used to place the other end of the shunt In the most proximal side of the CCA. A longitudinal arteriotomy was then performed alongside the occluded region (Figure 3A). The examination revealed thrombosis within the common carotid artery, accompanied by an underlying intimal flap. The damage done to the vessel was limited only to the vessel intima, without a subintimal involvement. A direct thrombectomy was executed, and the intimal flap was fixated with 6-0 nylon sutures. The arteriotomy was subsequently closed using a double-layered autologous saphenous vein patch (Figure 3B).

Upon de-shunting the artery, a restoration of normal pulsatile blood flow was observed in all the carotid arteries, followed by Doppler ultrasound confirmation.

Follow-up and outcomes

**Figure 4:**
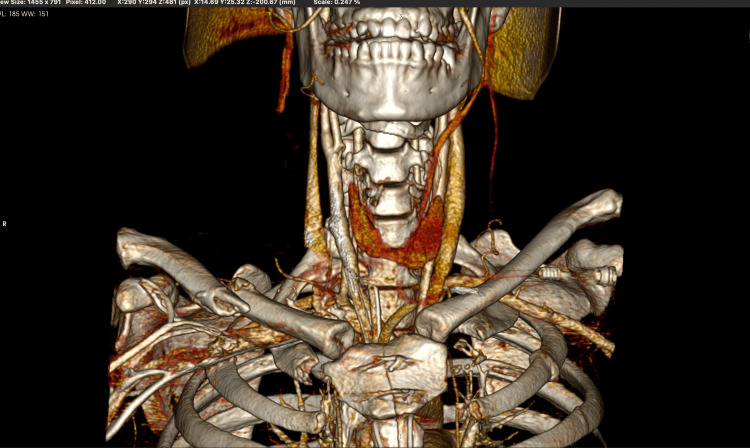
A postoperative 3D reconstruction based on a CT presenting complete revascularization of the right CCA CCA: common carotid artery

Post-surgery, the patient had an uneventful postoperative and recovery period without any major adverse events and was discharged on the thirteenth postoperative day on a single antithrombotic agent - clopidogrel. A follow-up exam conducted three months post-surgery showed significant clinical improvements: the patient was able to walk on his own without a cane. Unfortunately, no improvement was found regarding the loss of vision and muscle power in the left arm. A CT angiography performed at three months post-surgery showed a patent arterial reconstruction with improved flow through the right internal carotid and middle cerebral arteries (Figure 4). The Doppler sonography at the third month showed improved volume flow in the right middle cerebral artery.

## Discussion

Injuries to the carotid artery occur in blunt trauma as a result of either a primary insult (eg, from direct impact or laceration from the surrounding bony structures) or by shearing forces on the vessel that are the consequence of hyperflexion, extension, or rotation of the neck [9].

Blunt traumatic injuries to the carotid artery, particularly those confined to the CCA, are uncommon [10,12,13]. Guidelines on carotid disease management do not make any differentiation between spontaneous and traumatic dissection or occlusion [1], and there is still a debate on how this different entity should be treated without specific high-grade, evidence-based guidelines. Biffl et al. introduced the most commonly acknowledged grading system for blunt cerebrovascular injury (BCI), which relies on imaging findings and is commonly referred to as the Denver criteria [14]. These five grades are as follows: Grade I - disruption inside the vessel that results in a narrowing of the lumen by less than 25%; Grade II - dissection or intramural hematoma causing greater than 25% stenosis; Grade III - comprises pseudoaneurysm formation; Grade IV - total vessel occlusion; Grade V - describes vessel transection with extravasation [14]. Treatment decisions have predominantly relied on these criteria. Grade I and II injuries, or when a lesion is inaccessible, typically involve management through antithrombotic therapy alone, including anticoagulants like heparin or antiplatelet agents such as aspirin or clopidogrel. For Grade III injuries or accessible lesions, intervention is often warranted. Nevertheless, ongoing challenges persist in refining the optimal duration of therapy, determining intervention indications, and establishing the timing of intervention. Although most patients diagnosed with BCI are treated conservatively with systemic anticoagulation or antiplatelet therapy, a small subset may benefit from operative intervention either through an open surgical or endovascular approach [15,16].

Due to the high rates of stroke in patients with grade IV lesions, intervention is advocated, whether surgical or interventional radiological [17]. In addition, a large-scale multicenter study done by Jacob-Brassard et al. described that carotid intervention in patients suffering from neurological deficits is associated with an increased likelihood of home discharge [10]. In all grades, low or high, antithrombotic or anticoagulant therapy is the standard of care [1].

In our presented case, the patient suffered from a Grade IV complete traumatic occlusion of the CCA with a possible duration of almost a month and a clear neurologic deficit. All these factors weighted in favor of choosing the operative approach. Many authors, based on extensive studies involving large cohorts, recommend surgical repair for accessible grade IV blunt cerebrovascular arterial injuries (BCAIs) [1,14]. Because of the total occlusion of the CCA and the accompanying neurological findings, the young age of the patient, and the persisting neurological deficit despite the best medical treatment, it was considered that leaving this young patient on conservative treatment alone would not be the best option due to the clinical evidence for poor collateralization. Our decisions are based entirely on the above-mentioned clinical presentation and judgment that restoration of normal brain blood supply is the best option for the treatment of such cases.

While the endovascular approach may provide a solution for surgically inaccessible lesions [10], such treatment was not considered appropriate due to the total occlusion of the CCA. Therefore, thrombectomy, followed by a primary repair of the intimal laceration and a double-layered patch angioplasty were considered to provide maximal benefit to the current patient, and in fact, an excellent result was obtained in terms of symptomatic regression. Keeping in mind the potential for neointimal hyperplasia in the future, we deliberately chose not to extend the arteriotomy and the following patch angioplasty to the ICA, giving the patient a decent lumen size in the CCA to further minimize the risk of future stenosis.

We’ve opted for a shunt placement because the ICA was still perfused through the retrograde blood flow from the ECA.

Another argument could be made that the surgical repair alone acts as a prevention of further ischemic stroke. As emphasized by Okada et al., the onset of neurological symptoms following trauma is typically reported to occur between one month and one year. However, longer durations, ranging from 2 to 12 years, have also been documented [13]. In our case, the main indication for surgical repair was an improvement in cerebral perfusion and prevention of future thromboembolic sources from the injured CCA. As there was no damage to the vessel intima, we opted not to perform an endarterectomy. In cases of severe damage to the vessel wall, resection and replacement of the involved segment may become necessary. Given the patient's young age and lack of comorbidities, we’ve decided to use an autologous saphenous vein, as its intact endothelium significantly reduces the risk of restenosis and thrombosis of the reconstruction and gives the best long-term patency. The vein was additionally modified to be used as a double-layered vein patch to further reduce the risk of future aneurysmatic dilatation. There is limited data in the literature focused on the timing of the intervention and most of it is focused on the endovascular approach. A study by Blitzer et al. highlighted that there was a clear and pronounced mortality benefit when delaying intervention, for how long exactly is yet to be determined [9].

## Conclusions

Our case underscores the importance of a multidisciplinary approach in the management of BCI, particularly in cases of complete vessel occlusion with neurological deficits. The successful outcome achieved in our patient, following prompt recognition and surgical intervention guided by the Denver criteria, highlights the potential efficacy of surgical repair in select cases where endovascular approaches may not be feasible or appropriate. While conservative management with antithrombotic therapy remains a cornerstone in BCI management, surgical intervention should be considered in patients with severe neurological deficits or high-grade injuries, as demonstrated in our case. The choice of surgical technique, including thrombectomy, primary repair, and patch angioplasty, should be tailored to individual patient characteristics and injury severity, to optimize cerebral perfusion and prevent further ischemic events. Long-term follow-up and continued surveillance are essential to assess the durability of arterial reconstruction, monitor for recurrent thromboembolic events, and evaluate neurological outcomes. Further research is needed to refine management algorithms, clarify indications for surgical intervention, and elucidate the comparative effectiveness of surgical versus endovascular approaches in BCI.

In conclusion, this case adds to the growing body of literature on BCI management and underscores the critical role of interdisciplinary collaboration and individualized treatment strategies in optimizing outcomes for patients with this rare but potentially devastating vascular injury.
